# The association between adult-type hypolactasia and symptoms of functional dyspepsia

**DOI:** 10.1590/1678-4685-GMB-2017-0015

**Published:** 2018-01-22

**Authors:** André Castagna Wortmann, Daniel Simon, Luiz Edmundo Mazzoleni, Guilherme Becker Sander, Carlos Fernando de Magalhães Francesconi, Débora Dreher Nabinger, Camila Schultz Grott, Tássia Flores Rech, Felipe Mazzoleni, Vagner Ricardo Lunge, Laura Renata de Bona, Tobias Cancian Milbradt, Themis Reverbel da Silveira

**Affiliations:** 1 Postgraduate Program in Sciences of Gastroenterology and Hepatology, Universidade Federal do Rio Grande do Sul (UFRGS), Porto Alegre, RS, Brazil.; 2 Human Molecular Genetics Laboratory, Universidade Luterana do Brasil (ULBRA), Canoas, RS, Brazil.; 3 Division of Gastroenterology, Hospital de Clínicas de Porto Alegre (HCPA), Porto Alegre, RS, Brazil.; 4 Molecular Diagnostic Laboratory, Universidade Luterana do Brasil (ULBRA), Canoas, RS, Brazil.

**Keywords:** bloating, dyspepsia, gastrointestinal diseases, lactose intolerance, single nucleotide polymorphism

## Abstract

Functional dyspepsia and lactose intolerance (adult-type hypolactasia, ATH) are common conditions that may coexist or even be confounded. Their clinical presentation can be similar, however, lactose intolerance does not form part of the diagnostic investigation of functional dyspepsia. Studies on the association between functional dyspepsia and ATH are scarce. This study aimed to evaluate whether ATH is associated with symptoms of functional dyspepsia. Patients fulfilling the Rome III diagnostic criteria for functional dyspepsia underwent genetic testing for ATH. Dyspeptic symptoms were evaluated and scored according to a validated questionnaire. The diagnostic criteria for ATH was a CC genotype for the -13910C/T polymorphism, located upstream of the lactase gene. The mean scores for dyspeptic symptoms were compared between patients with ATH and those with lactase persistence. A total of 197 functional dyspeptic patients were included in the study. Mean age was 47.7 years and 82.7% patients were women. Eighty-eight patients (44.7%) had a diagnosis of ATH. Abdominal bloating scores were higher in ATH patients compared to the lactase persistent patients (*P*=0.014). The remaining dyspeptic symptom scores were not significantly different between the two groups. The study results demonstrate an association between ATH and bloating in patients with functional dyspepsia.

Dyspepsia affects more than 20% of the world population (Ford *et al.*, 2015). It corresponds to a group of digestive symptoms with heterogeneous pathophysiology. Most patients with dyspeptic symptoms have functional dyspepsia (FD), which means no underlying cause was identified during diagnostic evaluation (Talley and Ford, 2015). According to the Rome IV criteria, FD is characterized by one or more of the following: postprandial fullness, early satiation, epigastric pain and epigastric burning, which are unexplained after routine clinical evaluation. FD includes two subcategories: postprandial distress syndrome (PDS) that is characterized by meal-induced dyspeptic symptoms, and epigastric pain syndrome (EPS) that does not occur exclusively postprandially; the two subgroups can overlap (Stanghellini *et al.*, 2016). The current definition, specifically for FD, represents a slight modification from the previous Rome III criteria, with the purpose of improving the specificity of definitions of syndrome's symptoms. Rome IV emphasizes PDS and EPS subtypes rather than focusing on the syndrome as a whole (Talley *et al.*, 2016).

The majority of the world's population has deficiency of lactase activity in adulthood (Mattar *et al.*, 2012). Lactase non-persistence after infancy results from a genetically determined condition known as adult-type hypolactasia (ATH), which is the main cause of lactose intolerance (Misselwitz *et al.*, 2013). Single nucleotide polymorphisms (SNPs) located upstream of the lactase gene are associated with ATH (Enattah *et al.*, 2002). The most important SNPs are known as -13910C/T and -22018G/A, and genetic testing is currently available as a non-invasive method to diagnose this condition (Rasinperä *et al.*, 2004; Högenauer *et al.*, 2005; Krawczyk *et al.*, 2008; Pohl *et al.*, 2010; Misselwitz *et al.*, 2013). The most common symptoms of lactose intolerance are abdominal bloating, diarrhea, flatulence, nausea and vomiting (Levitt *et al.*, 2013). However, these symptoms are not specific and may be caused by other conditions, such as functional gastrointestinal disorders (FGIDs) (Drossman, 2006; Jellema *et al.*, 2010).

Lactose intolerance and FGIDs are common conditions that may coexist or even be confounded. There may be some overlap between the symptoms of lactose intolerance and functional dyspepsia. A few particular symptoms, such as abdominal bloating, nausea and vomiting, may be attributed to both conditions (Drossman, 2006; Jellema *et al.*, 2010; Levitt *et al.*, 2013). Indeed, the pathophysiology of FD is complex, heterogeneous and not yet completely understood (Vanheel and Farré, 2013). More recently, the role of dietary factors in FD has been increasingly recognized (Feinle-Bisset *et al.*, 2004, Feinle-Bisset and Azpiroz, 2013; Shepherd *et al.*, 2013).

Conceptually, the diagnosis of functional dyspepsia implies a lack of evidence of any organic, systemic or metabolic disease that is likely to explain the symptoms (Stanghellini *et al.*, 2016). However, investigation of lactose intolerance (which is caused by adult-type hypolactasia) does not currently form a part of the diagnostic testing for dyspepsia. In a recent review of the role of diet in FD, Feinle-Bisset and Azpiroz (2013) stated the importance of recognizing potential organic causes for symptom induction, including gluten and lactose intolerance and food allergies. Data on the association between lactose intolerance and functional dyspepsia are scarce (Mishkin *et al.*, 1997; Wilder-Smith *et al.*, 2013). Therefore, this potential association needs additional investigation.

The aim of the present study was to evaluate whether adult-type hypolactasia is associated with symptoms of functional dyspepsia.

This study was nested in the HEROES trial (Helicobacter Eradication Relief of Dyspeptic Symptoms trial; ClinicalTrials.gov No. NCT00404534) (Mazzoleni *et al.*, 2011). This was a randomized, double-blind, placebo-con-trolled, clinical trial carried out in a single academic hospital (Hospital de Clínicas de Porto Alegre). In summary, patients with dyspeptic symptoms were recruited from referrals, from primary care settings, and through advertising media. Patients of either sex aged 18 years or more were included if they had a diagnosis of functional dyspepsia according to the Rome III criteria. Symptoms must have been present for more than six months, with at least one episode per week of epigastric pain, burning, discomfort, postprandial fullness, or early satiety, during the previous three months. All patients underwent an esophagogastroduodenoscopy with gastric and duodenal biopsies, and only those who tested positive for *Helicobacter pylori* (from both histopathological examination and urease test) were included in the trial. Patients with findings suggestive of organic diseases, such as cancer of the upper gastrointestinal tract, erosive esophagitis, peptic ulcer disease and/or celiac disease were excluded. Patients with irritable bowel syndrome (IBS) were also excluded. Other exclusion criteria have been previously detailed (Mazzoleni *et al.*, 2011).

Dyspeptic symptoms were evaluated through a previously structured and validated questionnaire (Porto Alegre Dyspeptic Symptoms Questionnaire - PADYQ) (Sander *et al.*, 2004). This 11-item instrument assesses the three most important symptoms of FD (upper abdominal pain, abdominal bloating and early satiety) and also nausea and vomiting, during the preceding 30 days. The total score ranges from 0 (absence of symptoms) to 44 (severe symptoms). This procedure enables evaluation of the severity of each symptom through its frequency, intensity and duration. According to the predominant symptoms, patients were either considered to have PDS (bothersome postprandial fullness and/or early satiation, and possibly upper abdominal bloating or postprandial nausea or excessive belching) or EPS (epigastric pain or burning, and supporting criteria, as stated previously). In the present study, we considered exclusively the clinical evaluation of symptoms of FD performed at baseline in the HEROES trail.

All patients had blood samples stored, which were used for DNA extraction and subsequent analysis of ATH-associated polymorphisms. The local Institutional Review Board approved the study protocol, and informed consent was obtained from all patients (including permission for genetic testing) (protocol number GPPG-HCPA 100473).

DNA extraction from the blood samples was followed by polymerase chain reaction (PCR) amplification. Genotyping of the -13910C/T SNP (rs4988235) was performed by DNA sequencing, as described by Ingram *et al.* (2007). The diagnostic criterion for ATH was a CC genotype at SNP -13910C/T. A CT or TT genotype indicated the absence of ATH, which corresponds to lactase persistence. This choice is justified as this SNP has a 100% penetrance and is considered the most important ATH-associated SNP (Enattah *et al.*, 2002; Misselwitz *et al.*, 2013).

Quantitative data were described as means and standard deviations, and qualitative data as frequencies and percentages. The allele frequencies were determined by direct counting of the alleles, and deviations from the Hardy-Weinberg equilibrium were evaluated by a chi-square test. Mean age was compared between groups by Student's *t*-test. The mean PADYQ scores for upper abdominal pain, abdominal bloating, early satiety, nausea and vomiting were compared between patients classified as having ATH and those having lactase persistence by the Mann-Whitney U test. Categorical variables were compared using the chi-square or Fisher's exact tests, as appropriate. A *p* value of < 0.05 was considered significant. Statistical analyses were performed using PASW 18.0 (SPSS Inc., Chicago, USA).

Of 404 patients with functional dyspepsia and positive for *H. pylori* who were included in the HEROES Trial (Mazzoleni *et al.*, 2011), 197 consented to participate in this nested study aiming the analysis of the lactase gene polymorphism. Table 1 shows the demographic and clinical characteristics of the 197 patients with functional dyspepsia enrolled in the present study. The mean age was 47.7 ± 11.9 years and 163 (82.7%) were female. The mean PADYQ total score was 19.9 ± 7.1. A total of 104 (52.8%) patients had predominant symptoms categorized as PDS and 93 (47.2%) as EPS.

**Table 1:** Demographic and clinical characteristics of 197 patients with functional dyspepsia according to lactase activity status (ATH *versus* lactase persistent).

Variable	Total (n=197)	ATH* (n=88)	Lactase persistent (n=109)	*p*
Age, mean ± SD (years)	47.7 ± 11.9	47.4 ± 11.1	48.0 ± 12.6	0.700
Female, n (%)	163 (82.7)	74 (84.1)	89 (81.7)	0.652
Race (white), n (%)	167 (84.8)	71 (80.7)	96 (88.1)	0.151
Coffee drinker, n (%)	131 (66.5)	62 (70.5)	69 (63.3)	0.290
Smoking status, n (%)				0.183
*Never*	113 (57.4)	56 (63.6)	57 (52.3)	
*Current/Former*	84 (42.6)	32 (36.4)	52 (47.7)	
Alcohol intake, n (%)				0.428
*Never*	170 (86.3)	73 (83.0)	97 (89.0)	
*Current/Former*	27 (13.7)	15 (17.0)	12 (11.0)	
Duration of dyspepsia > 5 years, n (%)	94 (47.7)	44 (50.0)	50 (45.9)	0.286
Functional dyspepsia categories, n (%)				0.876
*Postprandial distress syndrome*	104 (52.8)	47 (53.4)	57 (52.3)	
*Epigastric pain syndrome*	93 (47.2)	41 (46.6)	52 (47.7)	

* ATH: Adult-type hypolactasia

According to the -13910C/T genotyping, 88/197 patients (44.7%) had ATH. The genotype frequencies of the -13910C/T polymorphism were as follows: CC, 88/197 (44.7%); CT, 89/197 (45.2%); and TT, 20/197 (10.1%). The frequency of the T allele was 32.7%. No deviation from the Hardy-Weinberg equilibrium was observed (*p*=0.718). Seventy-one (80.7%) patients with ATH were white, compared to 96 (88.1%) of the lactase persistent patients (*p*=0.15). Similarly, there were no significant differences in other demographic and clinical characteristics between the two groups (Table 1).

The results of the comparison between the scores for dyspeptic symptoms and lactase activity status are shown in Table 2. The total score for dyspeptic symptoms (PADYQ total score) was similar between patients with ATH (19.94 ± 6.32) and lactase persistence (18.31 ± 7.67) (*p*=0.134). However, comparison of the mean score for each symptom between the two groups showed bloating to be significantly higher in the ATH group (9.06 ± 2.55), compared to the lactase persistent group (8.32 ± 2.71) (*p*=0.014). In addition, although no statistically significant difference was seen, there was a tendency for more symptoms of nausea in the ATH group (*p*=0.063). Symptoms of epigastric pain, early satiety and vomiting had similar mean scores between the two groups. Similarly, there were no statistically significant differences in the frequency of ATH between FD subtypes (Table 1).

**Table 2:** Total and individual symptom scores (PADYQ) according to lactase activity status (-13910C/T SNP genotypes).

PADYQ* score	ATH**-13910 CC (n=88)	Lactase persistent-13910 CT + TT (n=109)	*p*
Upper abdominal pain	7.17 ± 3.31	7.06 ± 3.59	0.867
Nausea	4.85 ± 3.94	3.73 ± 4.06	0.063
Vomiting	0.37 ± 0.73	0.41 ± 0.97	0.458
Abdominal bloating	9.06 ± 2.55	8.32 ± 2.71	**0.014**
Early satiety	2.06 ± 1.55	1.96 ± 1.50	0.634
Total score	19.94 ± 6.32	18.31 ± 7.67	0.134

Data are presented as the mean ± standard deviation or number (percentage).

* PADYQ: Porto Alegre Dyspeptic Symptoms Questionnaire (Sander *et al.*, 2004)

** ATH: Adult-type hypolactasia

The present study evaluated whether adult-type hypolactasia is associated with symptoms of functional dyspepsia. Almost half the patients with FD in the sample were lactase deficient (diagnosed as ATH according to genetic testing). The frequency of ATH in our study is in accordance with the prevalence rates previously described in Brazilian populations (Bernardes-Silva *et al.*, 2007; Mattar *et al.*, 2009; Friedrich *et al.*, 2012). Our results showed that functional dyspepsia patients with ATH had higher bloating scores in comparison to those with lactase persistence. The remaining dyspeptic symptom scores did not differ significantly between these two groups.

A homogeneous sample of patients with FD was analyzed. All patients underwent a thorough evaluation in order to establish a diagnosis of FD. The rigorous diagnostic evaluation performed, including an upper gastrointestinal endoscopy with gastric and duodenal biopsies, aimed at excluding from the sample any patients with other overlapping or confounding conditions. Clinical evaluation was performed using a structured, validated questionnaire, giving an effective and reproducible assessment of FD symptoms (Sander *et al.*, 2004). This instrument allowed a thorough evaluation of the symptoms (intensity, frequency and duration), both individually and as a whole. Lactase activity status was assessed through molecular analysis and patients were classified either as ATH or lactase persistent (non-ATH), according to the genetic test result. ATH is ultimately the major cause of lactose intolerance and an excellent genotype-phenotype correlation has been reported for the -13910C/T SNP (Rasinperä *et al.*, 2004; Högenauer *et al.*, 2005; Ridefelt and Håkansson, 2005; Anthoni *et al.*, 2007; Bulhões *et al.*, 2007; Usai Satta *et al.*, 2008; Pohl *et al.*, 2010). Thus, this non-invasive diagnostic approach was performed in order to investigate the association between adult-type hypolactasia and symptoms of functional dyspepsia.

Food ingestion is associated with symptom onset or exacerbation in a significant proportion of patients with FGIDs (Feinle-Bisset and Azpiroz, 2013). The role of food has been more extensively studied in IBS. A consistent body of evidence has linked FODMAPs (fermentable, oligo-, di-, monosaccharides, and polyols) and IBS, as reviewed by Shepherd *et al.* (2013). In particular, the potential relevance of lactase deficiency in IBS symptoms has been a matter of debate for decades, yielding mixed results (Brandt *et al.*, 2009). Dietary factors also seem to be important in FD (Carvalho *et al.*, 2010; Feinle-Bisset and Azpiroz, 2013; Shepherd *et al.*, 2013; Goktas *et al.*, 2016). However, the role of the ingestion of milk and dairy products in symptoms of patients with FD is still unclear. Some authors have evaluated the frequency of lactose intolerance or malabsorption and ATH in patients with dyspeptic complaints (Mishkin *et al.*, 1997; Di Stefano *et al.*, 2009; Mattar *et al.*, 2009; Wilder-Smith *et al.*, 2013). Three of these studies have either heterogeneous samples (also including patients with FGIDs other than FD) or poorly characterized dyspeptic patients (Mishkin *et al.*, 1997; Di Stefano *et al.*, 2009; Mattar *et al.*, 2009). An observational study with a large sample conducted by Wilder-Smith *et al.* (2013) evaluated lactose intolerance and malabsorption in 606 functional dyspeptic patients. In total, 49.7% of these patients were classified as lactose intolerant. However, symptoms were not evaluated according to status of lactase activity. Additionally, none of these studies were specifically designed to evaluate FD symptoms in relation to ATH or lactose intolerance.

The similarity of some clinical aspects of FD and lactose intolerance provides a rational basis for the present investigation. In particular, bloating is a common and bothersome symptom that may form part of the clinical presentation of FGIDs, such as IBS and FD (Knill-Jones, 1985; Talley *et al.*, 1989; Lembo *et al.*, 1999; Longstreth *et al.*, 2006). Indeed, bloating is also considered one of the typical symptoms in the definition of lactose intolerance (Misselwitz *et al.*, 2013). Since genetic testing for ATH correlates well with lactase activity (Rasinperä *et al.*, 2004; Högenauer *et al.*, 2005; Pohl *et al.*, 2010; Bulhões *et al.*, 2007; Usai Satta *et al.*, 2008; Anthoni *et al.*, 2007; Ridefelt and Håkansson, 2005), it seems reasonable to consider the possibility of such an underlying pathophysiological mechanism to explain the finding of higher bloating scores among patients with lactase deficiency (ATH). In our understanding, the same may also explain a tendency of higher nausea scores in this group of patients. Besides, it would be unreasonable to find a pathophysiologic basis to explain symptoms like early satiety, epigastric and vomiting in the context of ATH. Thus, our observation that there is no association of ATH and total score for dyspeptic symptoms, and also early satiety, epigastric pain and vomiting individually, highlights the association of ATH and bloating observed in our patients with FD. Moreover, a possible bias due to the overlap between FD and IBS is unlikely, since patients with IBS were not included in our sample because of a rigorous initial diagnostic evaluation. Thus, the findings of the present study indicate the possible presence of a subgroup of so-called “functional dyspeptic” patients who have one identifiable organic mechanism, which relates to lactase deficiency, or ultimately, lactose intolerance.

Some limitations in this study must be considered. First, we acknowledge there may be some influence of *H. pylori* gastritis itself in the pathophysiological basis of bloating in the patients studied. Currently, Rome IV criteria define *H. pylori*-associated dyspepsia in those patients with long term sustained remission of symptoms after bacterial successful eradication. Since all recruited patients had *H. pylori* infection at inclusion in the HEROES trial, the symptoms of the patients studied could be, at least in part, related to *H. pylori* gastritis itself. Unfortunately, study design is limited to elucidate the complex interaction between these factors. Additionally, another limitation of the present study is the lack of dietary information regarding milk and derivatives ingestion. This is due to the fact that the present study was conceived after the HEROES study.

In conclusion, the findings of the present study demonstrate an association between adult-type hypolactasia and bloating in patients with functional dyspepsia. It is suggested that patients with functional dyspepsia who present with bloating should be evaluated for adult-type hypolactasia.
